# Impact of autologous blood transfusions on surface marker and microRNA profiles of urinary extracellular vesicles

**DOI:** 10.20517/evcna.2025.89

**Published:** 2025-11-28

**Authors:** Veronika Mussack, Michael W. Pfaffl

**Affiliations:** Animal Physiology and Immunology, School of Life Sciences Weihenstephan, Technical University of Munich, Freising 85354, Germany.

**Keywords:** Urine, EV, miRNA, blood doping, transcriptomics, RNA sequencing, NGS, RAAS

## Abstract

**Aim:** Autologous blood transfusions (ABT), especially those involving stored erythrocyte concentrates (ECs), are known to be misused as performance enhancers in recreational and competitive athletes. EC storage not only increases the release of extracellular vesicles (EVs), but also significantly alters the microRNA profiles. Since re-transfused EVs also appear in urine, this study was designed to evaluate whether the urinary EV-associated microRNA load could serve as a valuable indicator in the challenging detection of ABT.

**Methods:** Thirty healthy, recreationally active males were included and equally divided into three groups. The control group did not donate or receive any blood components. Group 1 donated about 500 mL of whole blood once, which was subsequently processed into ECs. These were stored for six weeks and then re-infused into the respective donor. Group 2 donated about 500 mL of whole blood twice, at an interval of two weeks. The obtained ECs were stored for six or four weeks, respectively, until parallel re-infusion. In all groups, urine samples were collected over three consecutive weeks before whole-blood donation to establish each individual’s baseline, as well as before re-transfusion and several hours and days afterward. Urine samples were processed and analyzed for general urinary health and creatinine levels. Urinary EVs were further isolated by immunoaffinity and characterized using transmission electron microscopy, fluorescence nanoparticle tracking analysis, and western blotting, as well as an established multiplexed bead-based flow cytometry method, followed by RNA isolation and in-depth small RNA profiling using next-generation sequencing and comprehensive data analyses.

**Results:** Urinary EVs presented with typical morphology of small EVs (< 200 nm) and an overall concentration of 8.79 ± 7.00 × 10^10^ particles/g U_Crea_ (urinary creatinine). Significant increases in urinary EV concentrations were detected up to three days after ABT. Apart from Alix, Syntenin, and tumor susceptibility gene 101 (TSG101), surface markers CD63, CD9, CD133/1, CD24, CD326, CD81, and CD31 were also shown to be highly abundant on urinary EVs. Impurities or contaminations were not detected. Cluster analysis based on surface markers showed a clear separation between the control and ABT group. Furthermore, microRNA profiling revealed 13 microRNAs differently regulated upon ABT with miR-155-5p, miR-320b, and miR-6869-5p being the most abundant.

**Conclusion:** This proof-of-concept study suggests an impact of ABT on the urinary EV-microRNA cargo and yields comprehensive findings into surface markers of urinary EVs. While the adoption of urinary EV-associated microRNAs in routine doping tests requires further exploration, these data serve as a valuable basis for a variety of subsequent investigations.

## INTRODUCTION

Blood transfusions are routinely used in surgeries involving high blood loss or in the treatment of patients with anemia. Among the various blood products, however, it is the reintroduction of stored erythrocyte concentrates (ECs) that is misused by high-performance athletes seeking to enhance their oxygen-carrying capacity^[1,2]^. The World Anti-Doping Agency (WADA) prohibits any form of blood doping, including EC infusions, to ensure equality in sports, and sanctions every rule violation^[3,4]^. While homologous blood transfusions, where the donor and recipient are different individuals, can be identified quite reliably^[5]^, the detection of autologous blood transfusions (ABT) remains a huge bottleneck in anti-doping, and valid verification of ABT in doping cases has so far proven virtually impossible^[6]^. In this light, the Athlete’s Biological Passport (ABP) was introduced as a powerful tool in the anti-doping repertoire to spot any non-physiological variation by, among other means, monitoring the longitudinal hematological profile of each individual athlete^[7]^. However, in many cases, unequivocal convictions of cheating athletes have relied primarily on whistleblowers^[8]^.

For this reason, current anti-doping research increasingly focuses on indirect detection of blood doping^[6]^. In initial investigations, analyzing an individual’s transcriptome appeared very promising to detect the misuse of growth-promoting substances or recombinant erythropoietin^[9-12]^. microRNAs (miRNAs), which are small 22-25 nucleotide long non-coding RNAs^[13]^, are among the most promising biomarker candidates in this and other fields, such as oncology and cardiovascular research. Physiologically, they exert a great regulatory impact on more than 60% of the protein-coding gene set by repressing messenger RNA at the post-transcriptional level^[14,15]^. Interestingly, it has already been shown that erythrocytes contain a diversity of miRNAs, and that their miRNA profiles significantly change during storage^[16-20]^. In addition, erythrocyte storage increases the release of extracellular vesicles (EVs)^[21,22]^, which are key intercellular communicators transferring a diverse cargo of bioactive compounds, including lipids, proteins, and various types of nucleic acids^[23-27]^. Vesicle-enclosed RNA is exceptionally well-protected from degradation and hence very promising in biomarker research^[28-31]^. Given their stability, accessibility via non-invasive procedures and sensitive quantification, miRNAs originating from urinary EVs (uEVs) have already been utilized for the detection of renal and urogenital diseases^[32-35]^. Despite the initial belief that EVs are too large for glomerular filtration, they were later shown to be eliminated via urine when reintroduced into the circulation^[36,37]^. However, the foregoing biomarker potential of uEV-miRNA has not yet been linked to reveal illegitimate ABT use in athletes.

Thus, we hypothesized that the re-transfusion of stored autologous ECs might lead to an increased turnover of aged erythrocytes, resulting in increased EV release and elimination via the urine with a significant shift in uEV-derived miRNA. These could act as a potent biomarker signature with the ability to discriminate ABT-doped and non-doped subjects in future field tests as a standalone marker or combined with the ABP. To this end, urine samples from healthy and recreationally active males were analyzed in consistent compliance with the “Declaration of Helsinki”^[38]^, WADA urine sampling guidelines^[39,40]^ and Minimal Information for Studies of Extracellular Vesicles (MISEV) 2018 and 2023 guidelines^[41,42]^. We separated uEVs by immunoaffinity and characterized them using transmission electron microscopy (TEM), fluorescence nanoparticle tracking analysis (fNTA), and western blot analysis (WBA), as well as an established multiplexed bead-based flow cytometry method (MBFCM), followed by RNA isolation and in-depth small RNA profiling using next-generation sequencing and comprehensive multivariate data analyses.

## METHODS

### Study subjects and design

The present study is based on previously published work, in which hematological parameters and whole blood miRNA profiles were monitored after ABTs^[43]^. The study was approved by the local ethics committee (Ludwig Maximilians-University of Munich, Germany, protocol #359-14) and conducted consistently according to the “Declaration of Helsinki” and the “Belmont Report”^[38,44]^. An upfront power analysis according to Bernhard Rosner^[45]^ was conducted to establish sample size. Each of the thirty male participants signed a written informed consent, including permission to use and publish the obtained data. The study population was randomized into three groups of ten individuals each. The control group neither donated blood nor received EC infusions; however, its sampling schedule was identical to that of the treatment groups to allow differentiation between ABT-dependent and non-ABT-dependent variations. Group 1 donated around 500 mL of whole blood once, which was subsequently separated into its components. The resulting ECs (approximately 240 mL) were stored at 4 °C for six weeks before re-infusion. Group 2 donated around 500 mL of whole blood twice, at an interval of two weeks. The corresponding ECs were processed, stored for six and four weeks, respectively, and re-infused in parallel (approximately 480 mL EC total).

**Figure 1:**
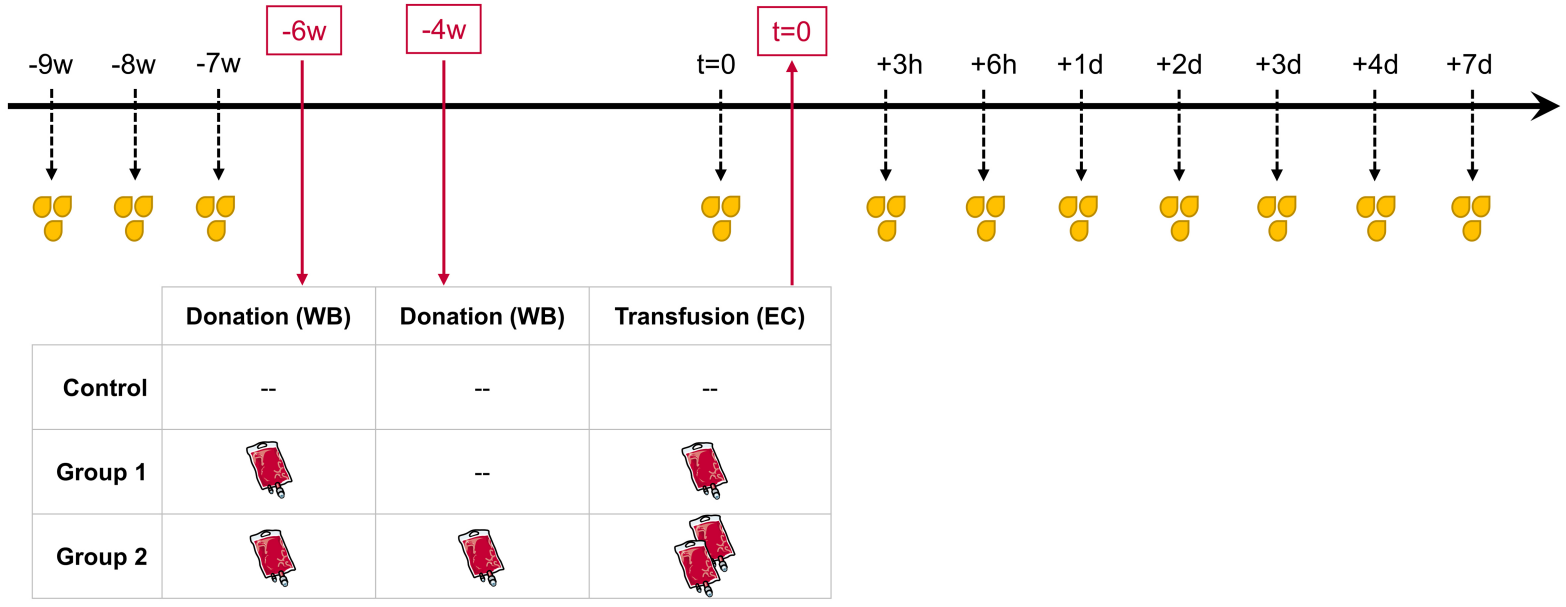
Longitudinal study design. All male subjects (*n* = 30) were randomly divided into three equal groups. WB bags (~ 500 mL, each) were donated six weeks prior to re-transfusion (-6 w) in Groups 1 and 2, and a second whole blood bag was donated in Group 2 four weeks prior to re-transfusion (-4 w). Depending on the grouping, either one (Group 1) or two (Group 2) processed and stored EC were re-transfused (*t* = 0). The Control group was analogously sampled for urine but without any blood donation or re-transfusion. Urine samples were collected before blood donation (-9 w, -8 w, -7 w) to create baseline measurements as well as shortly before re-transfusion (*t* = 0), and after re-transfusion (+3 h, +6 h, +1 d, +2 d, +3 d, +4 d, and +7 d) to identify transfusion-dependent variations. (www.https://smart.servier.com). WB: Whole blood; EC: erythrocyte concentrates; h: hours; d: days; w: weeks.

The present follow-up investigation aimed to examine changes in uEV-associated miRNA profiles following ABT. Urine samples were collected in three consecutive weeks (-9 w, -8 w, -7 w) before whole blood donation to establish each individual’s baseline and monitor physiological intra-individual variability. ABT-dependent variations were then analyzed in urine samples collected shortly before re-transfusion (*t* = 0) and at several hours (h) and days (d) afterward (+3h, +6h, +1d, +2d, +3d, +4d, +7d) [Figure 1].

### ABT

Preparation and re-transfusion of ECs was conducted according to Mussack *et al.*^[43]^. In brief, whole blood was obtained by puncturing the cubital vein using the sterile Composelect T3984-23 system (Fresenius, Germany) and a blood-mixing device (Compomixer M2; NPBI International B.V., Netherlands). Whole blood was leukocyte-depleted by filtration, followed by centrifugation (10 min, room temperature, 4,000 × *g*) to separate and concentrate erythrocytes prior to storage at 4 °C in PAGGS-M buffer (phosphate, adenine, guanine, glucose, sorbitol, and mannitol). Stored ECs were re-transfused via peripheral venous access after serological tests for blood typing and identity control, in strict adherence to the German Transfusion Law (BGBl. I 2007 S. 2169)^[46]^. Blood sampling and re-transfusion was conducted by medical doctors at the LMU University Hospital Großhadern (Ludwig Maximilians University of Munich, Germany).

### Urine sampling and pre-processing

Urine samples were collected in accordance with the WADA guidelines^[39,40]^ and following key recommendations of the position paper by the Urine Task Force of the International Society for Extracellular Vesicles^[47]^. Up to 150 mL of spontaneously voided urine samples (full void and random spot) were obtained. No additives were added at the time of collection. Subsequently, 10 mL of urine were instantly transferred into sterile vessels (Sarstedt AG & Co. KG, Germany) for creatinine determination and a general examination of urinary health. The remaining urine volume was kept in the dark at 4 °C for a maximum of 8 h, including a first centrifugation at 300 × *g* at 4 °C for 10 min to remove cells and a second centrifugation at 2,000 × *g* at 4 °C for 20 min to sediment larger particles and debris. The obtained supernatant was retained by pipetting. Next, aliquots of 10 mL cell-free urine each were prepared for either uEV separation and characterization, uEV phenotyping, or uEV separation and RNA analysis. The remaining cell-free urine was equally aliquoted as retention samples. All aliquots were immediately frozen at -80 °C and stored at -80 °C until further processing.

### Assessment of urine health and creatinine levels

Urine dipsticks were applied to confirm urine health using the Clinitek Novus (Siemens Healthineers, Germany) and Urisys 1800 (F. Hoffmann-La Roche, Switzerland) instruments based on an optical system and reflection photometry, respectively. As urinary creatinine levels (U_Crea_) strongly correlate to uEV concentration^[48,49]^, they were measured in all samples in an accredited external laboratory based on the Jaffé method^[50]^ (AU5800; Beckman Coulter, USA) and used to control and normalize for variances in urine concentration.

### Urinary extracellular vesicle separation

Stored urine samples were gently thawed overnight at 4 °C and vortexed extensively. The immunoaffinity-based Exosome Isolation Kit Pan, human (Miltenyi Biotec, Germany) was utilized to separate uEVs from 10 mL cell-free urine according to the manufacturer’s instructions, including an additional centrifugation step at 10,000 × *g* for 45 min at room temperature before an overnight sample incubation with CD9-, CD63- and CD81-antibody-labeled magnetic beads. uEVs were eluted in 100 µL of isolation buffer and either directly applied to RNA isolation or concentrated to a volume of around 50 µL by vacuum evaporation for uEV characterization by TEM, fNTA and WBA. The exact final volume for each sample was recorded and used for calculations in fNTA.

### Characterization of urinary EVs

To comply with the current MISEV2018 and MISEV2023 guidelines^[41,42]^, uEVs were characterized as reported by Mussack *et al.* and are described below^[51]^.

### TEM

Five µL of fresh uEV preparations were adsorbed onto formvar-carbon-coated nickel grids (200 mesh; Electron Microscopy Sciences, USA) for 5 min and negatively stained with 2% aqueous uranyl acetate for 5 min. Excess fluid was removed, and grids were dried at room temperature in the dark before capturing images at 80 kV using a Zeiss EM 900 transmission electron microscope (Zeiss, Germany) equipped with a wide-angle dual-speed 2KCCD camera.

### WBA

To assess the presence of EV-specific marker proteins and absence of contaminating proteins, in accordance with the MISEV2018 and MISEV2023 guidelines^[41,42]^, WBA was performed. Protein lysates were prepared using 15 µL of evaporated uEV isolates, which were incubated on ice in 2× radioimmunoprecipitation assay (RIPA) buffer supplemented with protease inhibitors (Sigma-Aldrich, Germany) for 15 min, with three 1-min bouts of ultrasonication every 5 min. According to the manufacturer’s recommendations, either reducing (5 min at 95 °C, 1× Laemmli buffer + ß-mercaptoethanol) or non-reducing conditions (5 min at 95 °C, 1× Laemmli buffer) were applied to investigate the different proteins. Eight µg of human embryonic kidney (HEK293) and 10 µg of HeLa cell lysates were equally treated as controls. Twenty µL of each uEV lysate were applied onto a 4%-12% sodium dodecyl sulfate-polyacrylamide gel electrophoresis (SDS-PAGE) gel (NuPAGE Bis-Tris, Invitrogen, USA) and separated at 100 V. PageRuler Prestained Protein Ladder (Fermentas/Thermo Scientific) was used as protein ladder. Subsequently, the gel was blotted onto a nitrocellulose membrane with a 0.45 µm pore size (GE HealthCare Life Sciences, USA) at 30 V for 75 min. Total protein composition was stained with Ponceau S [Supplementary Figure 1] before blocking the membrane for 1 h at room temperature in blocking buffer [1% skim milk powder in 1× TBST (Tris-Buffered Saline and Tween 20)]. Next, the membrane was incubated overnight at 4 °C with primary antibodies, followed by washing with blocking buffer and a 1-h incubation at room temperature with the corresponding secondary antibodies. Subsequently, the membrane was washed again with blocking buffer and phosphate-buffered saline (PBS). The Clarity Western ECL Substrate (Bio-Rad, UK) was applied before finally capturing protein signals with the Fusion FX6 system (Vilber, France). A list of the utilized primary and secondary antibodies is provided in Supplementary Table 1. As uEV isolation was based on immunoaffinity and the magnetic beads were coated with murine immunoglobulin G antibodies, background signals were recorded by incubating another membrane with an anti-mouse secondary antibody only [Supplementary Figure 2].

### fNTA

Particle diameters and concentrations were analyzed by fNTA using the ZetaView PMX110 (Particle Metrix, Germany), software version 8.04.02 SP, equipped with a 520 nm laser. The instrument was calibrated using 100 nm polystyrene beads and temperature-controlled at 24 °C. Measurement settings were adjusted as follows: shutter at 70, sensitivity at 95%, frame rate of 30 frames per second, trace length of 15, and two cycles of video capturing particles at eight to eleven different positions at high resolution. Post-acquisition parameters were set to a minimum brightness of 25, minimum size of 5 nm and maximum size of 10,000 nm. The fluorescence mode was used to discriminate between biological (e.g., EVs) and non-biological (e.g., magnetic beads from EV separation) particles. In these experiments, uEV samples were incubated with 5 µg/mL CellMask Orange Plasma Membrane Stain (Invitrogen, USA) for 30 min at 37 °C in the dark. Stained samples were further diluted in 1× PBS to reach the optimal device-specific measurement range of concentration. The obtained results were recalculated accounting for sample dilution and input volumes of uEV sample and urine^[52]^. Next, U_Crea_ was used to normalize for initial variances in urine dilutions^[49]^. To control for unspecific fluorescence signals and autofluorescence, buffer only, uEV samples only, magnetic beads only as well as beads plus membrane stain were evaluated in fluorescence mode, each resulting in no detectable signals. fNTA results were statistically analyzed and visualized as mean ± standard deviation (SD) using GraphPad Prism Software for Windows (version 8.4.1). One-way analysis of variance (ANOVA) was applied with correction for repeated measures and multiple comparisons by controlling the false discovery rate (FDR) according to Benjamini and Hochberg^[53]^. Adjusted *P*-values < 0.05 were considered significant.

### Multiplexed bead-based phenotyping of urinary EVs

The MACSPlex Exosome Kit, human (Miltenyi Biotec B.V. & Co. KG, Germany) was used for the comprehensive characterization of uEV surface marker profiles by MBFCM. Ten mL of thawed cell-free urine were used as starting material and pre-cleared by another centrifugation at 3,000 × *g* for 45 min to remove larger particles followed by an overnight incubation with capture antibodies and staining with a combination of CD9-, CD63- and CD81-allophycocyanin (APC) detection antibodies according to the manufacturer’s instructions. Median signal intensities were acquired with BD Accuri C6 (BD Biosciences, USA) at a flow rate of 100 µL/min. The FlowJo software, v10.6.1, was applied for gating and data processing^[54]^. Cell-free urine without antibody conjugation was examined for any autofluorescence or artifact generation [Supplementary Figure 3A]. Furthermore, a negative control containing only capture beads and MACSPlex buffer was included in the experimental setting to specify background signals [Supplementary Figure 3B], which were subtracted from the matched sample signals in subsequent data analysis. Moving forward, only signals above the corresponding isotype control were treated as valid. Signals below the isotype threshold were disregarded. To determine relative median fluorescence intensities (rMFI) and account for differences in urine densities, sample signals were normalized to U_Crea_. Surface marker distribution and abundance were shown as mean ± standard error. Log2 fold changes (log2FC) of U_Crea_-normalized signals were calculated using the respective individuals’ baseline levels (-9 w, -8 w, -7 w) as reference. Missing values were not replaced. Next, ABT-dependent dynamics in single uEV surface markers were examined for intra- and inter-group variations via two-way ANOVA with correction for repeated measures and multiple comparisons by controlling the FDR according to Benjamini and Hochberg^[53]^ using GraphPad Prism Software for Windows (version 8.4.1). Adjusted *P*-values < 0.05 were considered significant. To assess an overall ABT-dependent uEV surface marker pattern, row-scaled log2FC were explored by heatmap using Pearson correlation and the “pheatmap” package^[55]^ in R (version 3.6.0)^[56]^.

### RNA isolation and library preparation

Total RNA was extracted from uEV preparations using the miRNeasy Mini Kit (Qiagen, Germany) as specified in the manufacturer’s instructions except that the first RNA eluate was eluted a second time to yield more RNA. The RNA integrity number (RIN) was determined by capillary gel electrophoresis^[57]^. To this end, the Bioanalyzer 2100 (Agilent Technologies, Germany) in combination with the RNA 6000 Pico Kit was utilized. Afterwards, total RNA was stored at -80 °C until further processing.

Small RNA libraries were prepared from uEV-associated RNA as reported by Mussack *et al*.^[51]^. In brief, thawed total RNA was completely vacuum evaporated at room temperature using SC100 SpeedVac Concentrator (Savant instruments, India) in combination with the Diaphragm vacuum pump MD 4C (vacuubrand, Germany) and subsequently resuspended in 12 µL nuclease-free water. The NEBNext Multiplex Small RNA Library Prep Set for Illumina (New England Biolabs, USA) was used according to the manufacturer’s specifications. After purifying polymerase chain reaction (PCR) products with the Min Elute PCR Purification Kit (Qiagen, Germany), complementary DNA (cDNA) libraries were evaluated by capillary gel electrophoresis with the DNA 1000 Kit and the Bioanalyzer 2100 (Agilent Technologies). Barcoded cDNA libraries were pooled and applied to a 4% high-resolution agarose gel (MetaPhor Agarose, Lonza, USA) for size fractionation. Gel slices were cut at a miRNA-specific size range of 130-150 base pairs and purified via the MinElute Gel Extraction Kit (Qiagen) prior to quality and size assessment by capillary gel electrophoresis using the DNA High Sensitivity Kit (Agilent Technologies) and the Bioanalyzer 2100 (Agilent Technologies).

### Small RNA sequencing and data processing

Sequencing-by-synthesis of prepared cDNA libraries was performed on the HiSeq2500 instrument (Illumina, USA) via 50 cycles of single-end sequencing with the HiSeq Rapid SR Cluster Kit v2 combined with the HiSeq Rapid SBS Kit v2 (Illumina, USA). The obtained data were processed as described previously^[51]^. In brief, general sequencing quality was assessed based on the Quality Phred Score generated by the FastQC software (Babraham Bioinformatics, UK, Version 0.11.7)^[58]^. Sequences were trimmed for 3’ adaptor sequences using Btrim^[59]^. Reads without recognizable adaptor sequences or shorter than 16 nucleotides were rejected. After aligning trimmed and cleaned reads to sequences corresponding to ribosomal RNA (rRNA), transfer RNA, small nuclear RNA, and small nucleolar RNA, as provided by RNAcentral (version 9)^[60]^, remaining reads were mapped to human precursor miRNA sequences using miRBase, release 22^[61]^, and Bowtie^[62]^, while permitting for one mismatch. Unmapped reads were disregarded. A final read count table was generated by summing up all hits per miRNA sequence prior to importing it into the programming environment of R (version 3.6.2)^[63]^.

### Statistical analysis of sequencing data

The Bioconductor (Version 3.10) package “DESeq2” (Version 1.26.0) was applied to normalize miRNA reads^[64,65]^. Rlog-transformed data were examined for outlier samples and batch effects via principal component analysis (PCA) and hierarchical clustering analysis. Differential gene expression (DGE) analysis was conducted using DESeq2 by performing pairwise comparisons on DESeq2-normalized data at every single time point *vs*. baseline (-9 w, -8 w, -7 w) within each treatment or control group. miRNAs with |fold change (FC)| > 1.5 and a *P*-value < 0.05 were considered as significantly dysregulated. The overlap of significantly dysregulated miRNAs between the treatment and control groups was illustrated using Venn diagrams. Differences in log2FC between treatment and control groups were visualized and analyzed using two-way ANOVA in GraphPad Prism Software for Windows (version 8.4.1) regarding Benjamini-Hochberg adjusted *P*-values < 0.05 as significant. “ggplot2” was used to visualize the longitudinal profiles of individual miRNA levels^[66]^. In this proof-of-concept study, a static model was applied to identify unphysiological miRNA levels exceeding the 95% (z-score = |2|) or 99.7% (z-score = |3|) probability threshold.

## RESULTS

In this study, urine samples were collected from thirty healthy and recreationally active males (5.4 ± 2.0 h/week) (age: 27 ± 4 years; height: 182 ± 5 cm; weight: 79.9 ± 6.6 kg; BMI: 24.0 ± 1.9 kg/m^2^) alongside blood samples for whole blood miRNA analysis and hematological profiling according to Figure 1 and Mussack *et al.*^[43,67]^. The transfusion procedure was very well tolerated by all study subjects. A total of 321 samples were collected out of a maximum of 330 possible samples (*n* = 10 per group at 11 different time points). Urine samples demonstrated good health indicated by the absence of any blood cells, glucose, proteins, ketones, nitrite, and bilirubin. Furthermore, a mean specific gravity of 1,012.21 ± 7.00 g/L, pH at 6.27 ± 0.74, urobilinogen 0.30 ± 0.27 mg/dL, and U_Crea_ at 0.88 ± 0.69 g/L were detected.

### Comprehensive uEV characterization

**Figure 2:**
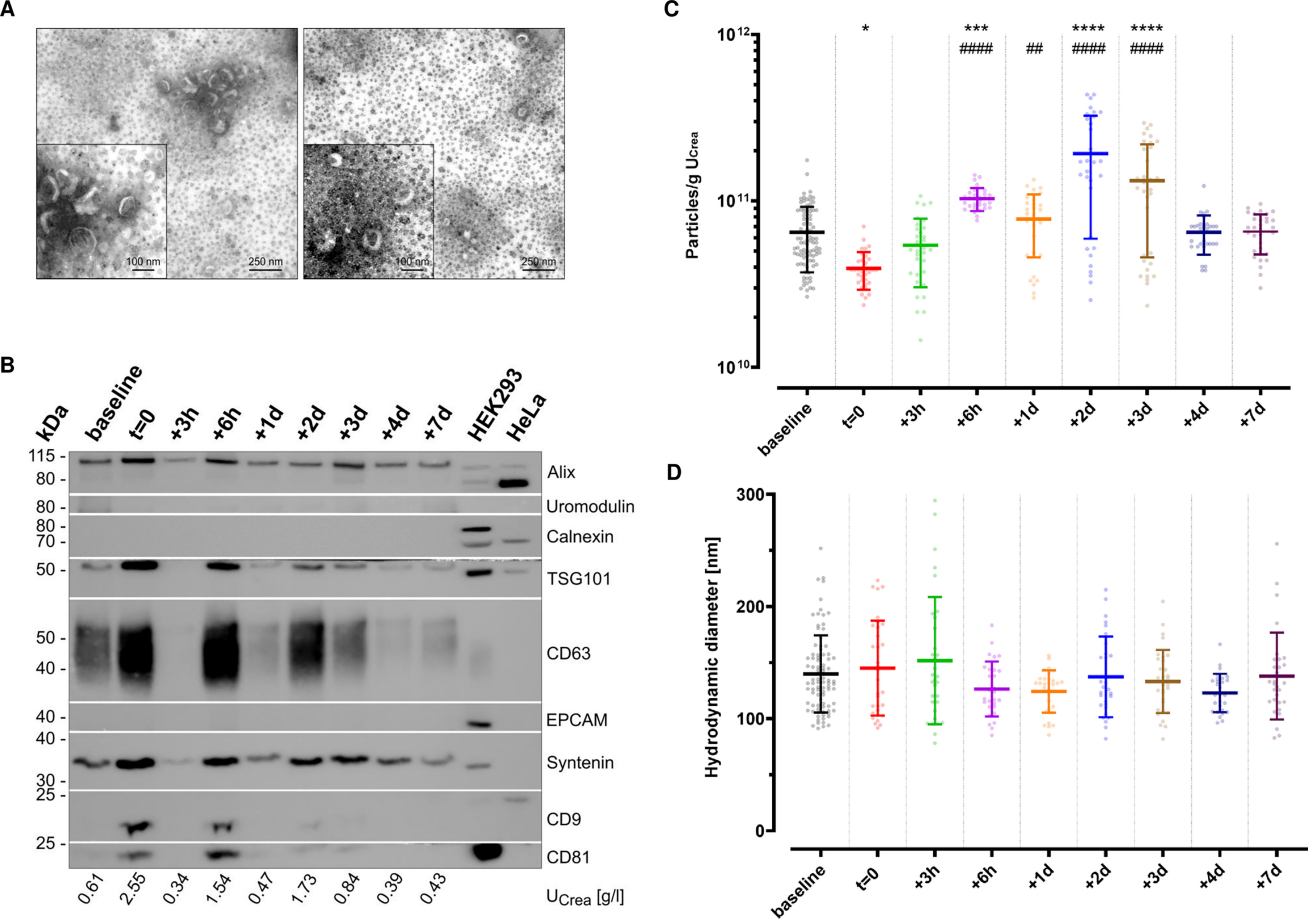
Characterization of uEVs separated by immunoaffinity. (A) TEM images of two representative samples illustrate desiccated vesicles (wide field: scale bar 250 nm; close-up: scale bar 100 nm). The omnipresent black dots indicate magnetic beads carried over from immunoaffinity-based enrichment. Brightness was adjusted for each other for better visibility using IrfanView software; (B) Western blot analysis of uEV-enriched eluates originating from one representative individual with different urinary dilutions, as indicated by the corresponding creatinine levels (U_Crea_). Images were cropped to the corresponding protein marker size range for a better overview. Baseline summarizes -9 w, -8 w, -7 w; (C) Fluorescence NTA (*n* = 3 of Group 2 with eight to 11 technical replicates each; baseline includes -9 w, -8 w, -7 w; mean ± SD; ^*^one-way ANOVA: adjust *P* < 0.05, ^***^P < 0.001, ^****^P < 0.0001 *vs*. baseline, adjusted ^##^*P* < 0.01, ^####^*P* < 0.0001 *vs*. *t* = 0) indicating particle concentration, normalized to input volumes and urinary creatinine levels (U_Crea_); and (D) hydrodynamic particle diameters; mean ± SD. ANOVA: Analysis of variance; uEV: urinary extracellular vesicle; TEM: transmission electron microscopy; U_Crea_: urinary creatinine; NTA: nanoparticle tracking analysis; SD: standard deviation; EPCAM: epithelial cell adhesion molecules; w: weeks.

As recommended in the MISEV guidelines^[41,42]^, uEVs enriched via immunoaffinity were characterized prior to any downstream analyses to assess the separation success. To this end, TEM was conducted to visualize uEV morphology [Figure 2A]. The applied negative staining resulted in typical artificial cup-shaped appearances in TEM, with heterogeneous diameters of desiccated vesicle-like structures around 100 nm. Apart from the kit-inherent magnetic beads, no other structures or contaminations were observed. WBA was performed to further screen for potential contaminating and uEV-specific proteins [Figure 2B]. As shown, the non-EV-specific structures Calnexin or Uromodulin were absent. Depending on the urinary dilution, as specified by each sample’s U_Crea_ levels, the presence of EV-specific protein markers - Alix, Syntenin, tumor susceptibility gene 101 (TSG101), CD9, CD63, and CD81 - could be confirmed at different signal intensities. No signals for epithelial cell adhesion molecule (EPCAM) were noticed in any of the samples. In general, urine concentration correlated with a higher probability of detecting tetraspanin signals in resulting uEVs as compared to more dilute urine. To complement uEV characterization, particle concentrations were assessed by fNTA. Across groups, an overall concentration of 8.79 ± 7.00 × 10^10^ particles/g U_Crea_ and overall hydrodynamic diameters of 156 ± 23 nm (median ± SD) and 137 ± 22 nm (mean ± SD) were observed. To assess the putative impact of ABT on uEV release, we next compared particle concentrations at the different sampling time points in a subset of three individuals of group 2 [Figure 2C]. While the urinary particle concentration was significantly lower at *t* = 0 compared to baseline (*P* = 0.0208), baseline levels were already recovered 3 h after re-transfusion of stored ECs. Remarkably, EC re-transfusion led to a continuously significant increase in urinary particle concentration compared to baseline levels (0.0004 < *P* < 0.0001) and *t* = 0 (0.0041 < *P* < 0.0001) from 6 h up to three days after re-transfusion. No significant time point-specific differences were observed for the hydrodynamic diameters of uEVs [Figure 2D].

**Figure 3:**
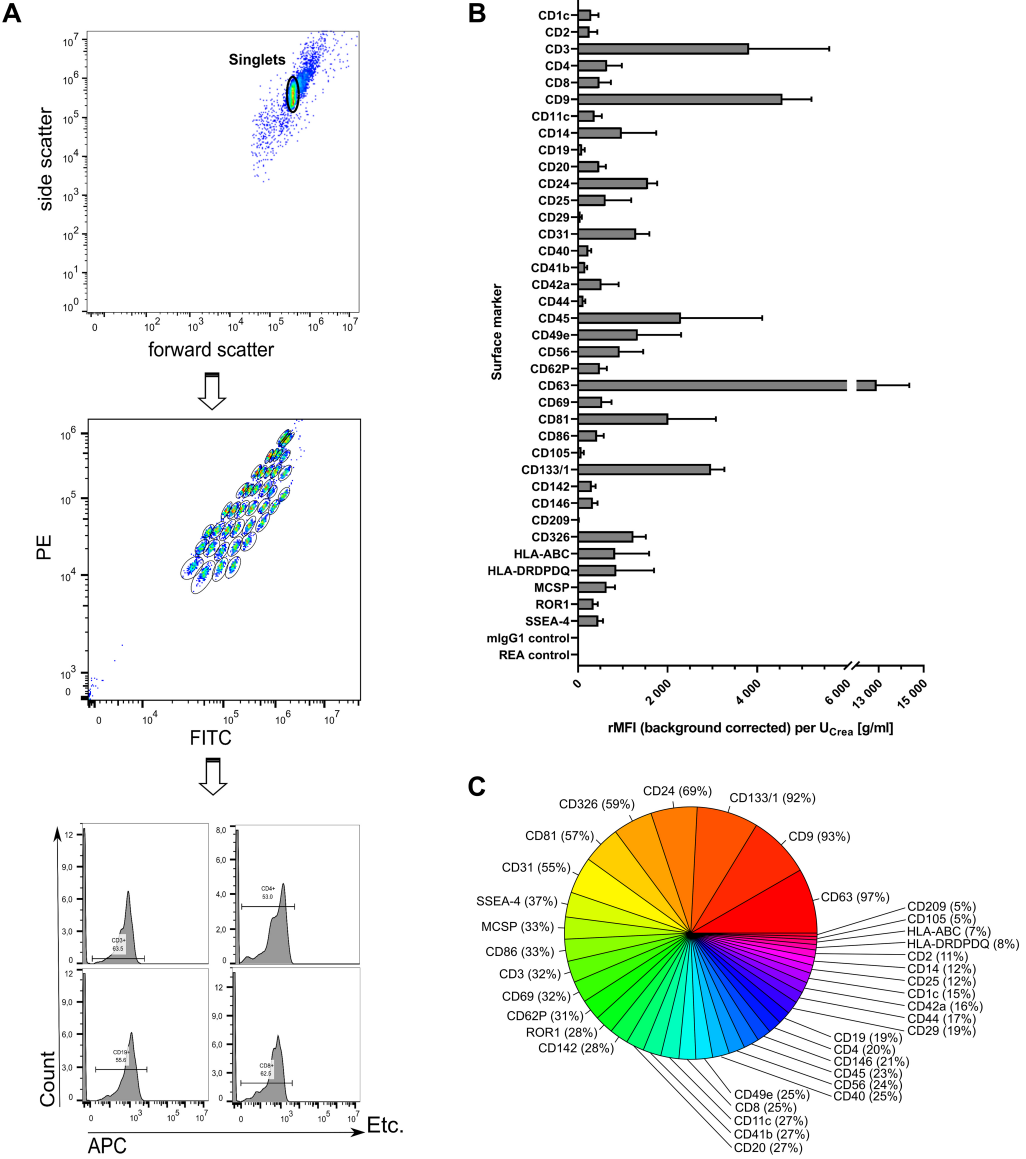
Multiplexed phenotyping of uEV surface markers. (A) Exemplary sample showing feature selection by including only the singlet population, gating on the different bead populations, and counting only APC-positive signals for each single bead population; (B) Overall surface marker distribution displayed as the rMFI after background correction and normalization to urinary creatinine levels (U_Crea_) displayed as mean ± standard error [overall *n* = 7; including control (*n* = 3) and group 2 (*n* = 4) at 11 time points each]; (C) Surface marker abundance illustrated as percentage of samples in which the respective marker was detected. uEVs: Urinary extracellular vesicles; rMFI: relative median APC fluorescence intensities; APC: allophycocyanin; U_Crea_: urinary creatinine.

To further explore uEV features including surface markers, MBFCM was utilized^[68]^. The capture bead population could be clearly gated separately and allowed for subsequent detection of APC-positive fluorescence signals of detection antibodies recognizing CD9, CD63, and CD81 epitopes [Figure 3A]. Thereby, stained CD9/CD63/CD81-positive uEVs were initially assessed for their overall diversity and abundance of numerous surface markers [Figure 3B and C]. After applying the isotype threshold, only seven surface markers were detected in more than 50% of samples (CD63, CD9, CD133/1, CD24, CD326, CD81, and CD31), while all others occurred less frequently (5%-37%). Almost every sample showed predominant signals for the tetraspanins CD63 and CD9 (12,906 ± 1,453 and 4,564 ± 641.1 rMFIs per U_Crea_ [g/mL] in 97% and 93% of samples, respectively). Notably, fluorescence signals detected for CD3 were the third highest with 3,818 ± 1,787 rMFIs per U_Crea_ [g/mL], present in about one third of all examined samples [Figure 3B]. Equally, CD45 appeared with 2,298 ± 1,814 rMFIs per U_Crea_ [g/mL], detectable in 23% of all samples.

### ABT-dependent surface marker variation on uEVs

**Figure 4:**
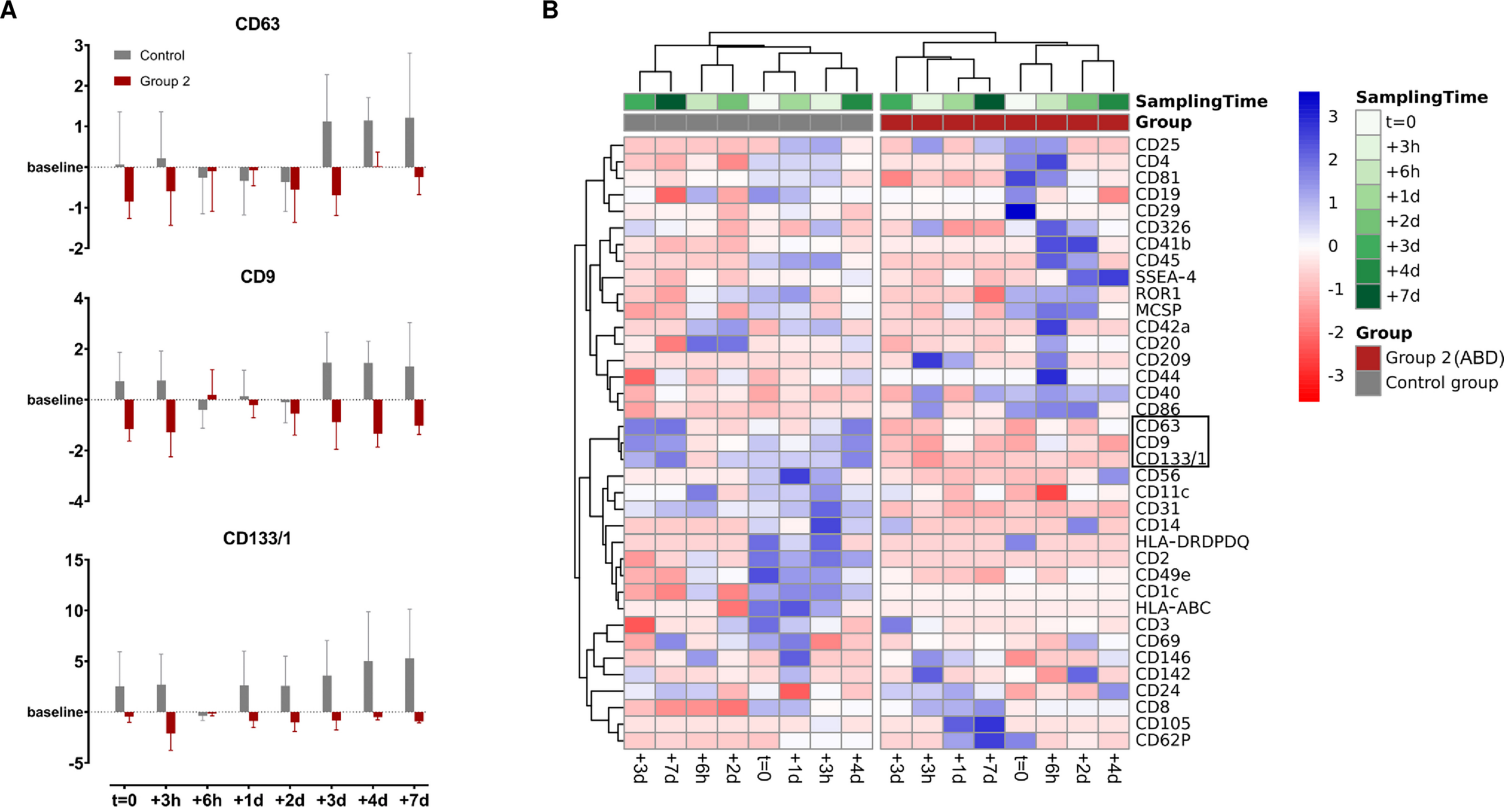
Visualization of the discriminative ability of surface marker dynamics. (A) Log2FC of CD63, CD9 and CD133/1 illustrated as mean ± standard error; (B) Heatmap clustering analysis of row-scaled log2FC were examined using Pearson correlation. (Control group, *n* = 3; Group 2, *n* = 4).

In the following, log2FC for each surface marker were calculated in relation to each individual’s baseline level to screen for potential ABT-dependent differences in the uEV surface marker profiles and evaluate whether ABT-dependent concentration differences also translate to the presence of different uEV subpopulations. To maximize the informational value of the analyses of the limited number of samples, only the most distinct groups - control and group 2 - were selected for intra- and inter-group comparisons. However, no significant differences were identified by ANOVA (0.5216 < *P* < 1), resulting from very high dynamics in surface marker profiles, missing marker detection, and a small sample size. Nevertheless, an almost universal trend of downregulation of CD63, CD9 and CD133/1 was discovered post ABT (log2FC < 0), while they appeared upregulated (log2FC > 0) in the control group as illustrated in Figure 4A. Remarkably, when all calculated log2FC were included in the heatmap cluster analysis based on Pearson correlation, with missing values also accounted for, a clear and complete separation between the control group and ABT-doped group 2 was observed [Figure 4B].

### ABT-dependent changes in miRNA profiles associated with uEVs

**Figure 5:**
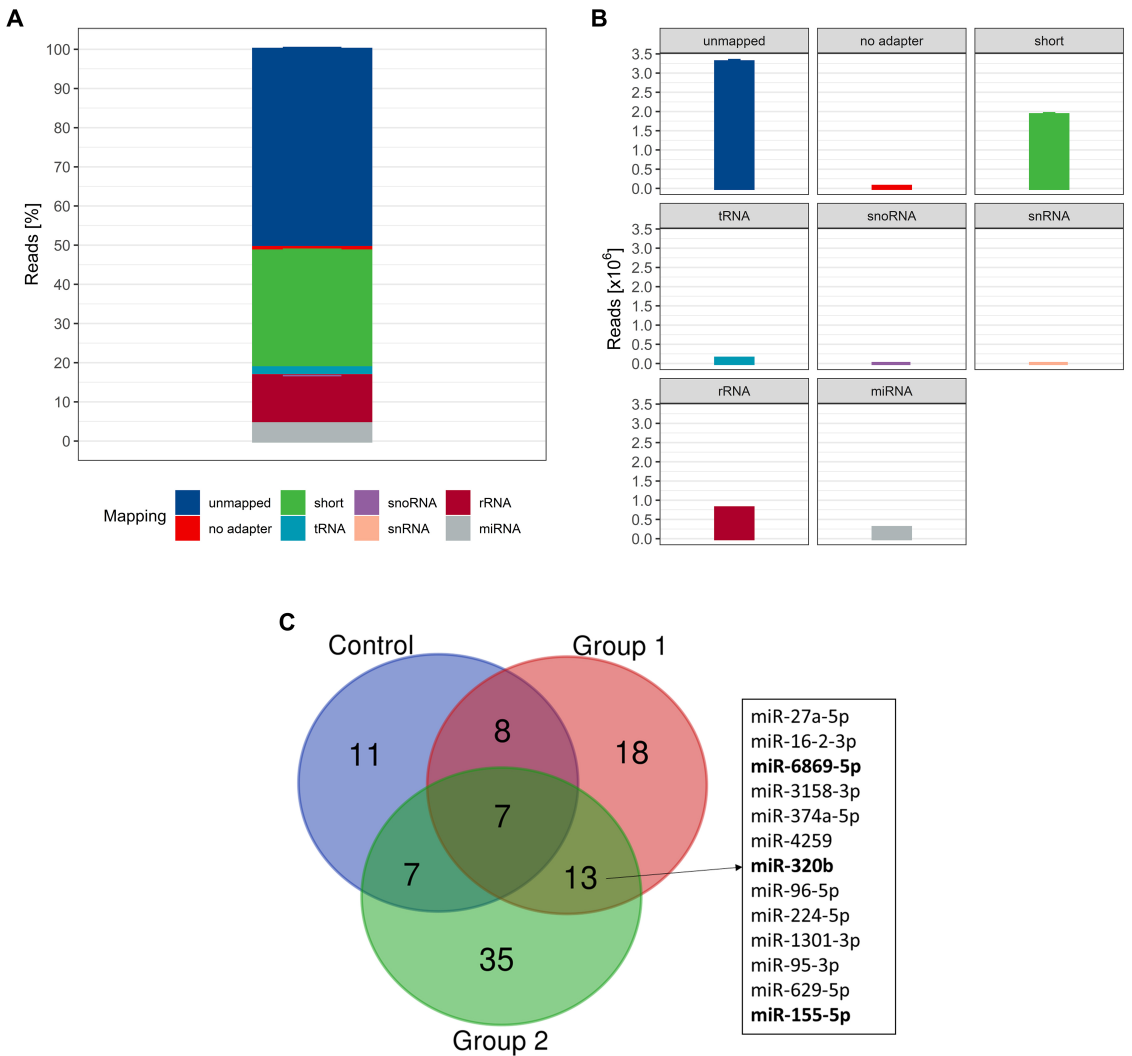
(A) Relative and (B) absolute mapping distribution. Data are shown as mean ± SD (*n* = 321); (C) Overlap of differentially expressed miRNAs; compared to intra-group baseline with |FC| > 1.5 and *P* < 0.05. Bold miRNAs were detected with DESeq2-normalized read counts > 20 (*n* = 320). SD: Standard deviation; miRNA: microRNA; FC: fold change; DESeq2: a normalization and differential expression analysis tool.

In the next step, we isolated and analyzed uEV-associated miRNAs to test our hypothesis of differential miRNA expression as a consequence of ABT. Initial quality control using capillary gel electrophoresis prior to small RNA sequencing (RNA-seq) library preparation revealed a mean RIN value of 1.5 ± 0.9 [Supplementary Figure 4], indicating the absence of cellular RNA - particularly the rRNA subtypes 18S and 28S, on which RIN calculation algorithms are based - and a mean total RNA yield of 2,590 ± 1,002 pg. Capillary gel electrophoresis of generated cDNA libraries resulted in peaks at 140 to 148 base pairs corresponding to the miRNA-specific size range. Quality control of sequencing success revealed a mean Phred Score of 39.64 (min: 27, max: 40), indicating an excellent base call accuracy of 99.99%. Overall, a total read count of 6.57 ± 1.5 × 10^6^ was obtained with the majority of reads being unmapped, short, or mapped to rRNA [Figure 5A and B]. 2.89% ± 0.88 × 10^5^ reads mapped to miRNAs, making up about 4.4% ± 0.8% of all reads. PCA and hierarchical clustering revealed one sample as an outlier which was disregarded from further analysis. No further outlier or batch effect was observed. In total, 1,972 distinct miRNAs were detected in the remaining 320 analyzed samples; however, only 186 miRNAs with at least one read count were detected in at least 75% of samples. Thus, these miRNAs were input into DGE analyses revealing 33 miRNAs with significant intra-group changes from baseline in the control group, 46 in group 1, and 62 in group 2 (*P* < 0.05; |FC| > 1.5). Only miRNAs differentially regulated in both treatment groups but not in the control group were further examined for their ABT-dependent profiles, resulting in 13 shared miRNAs. Three of these (miR-155-5p, miR-320b, and miR-6869-5p) featured DESeq2-normalized read counts > 20 and were thus selected for more detailed analysis [Figure 5C].

**Figure 6:**
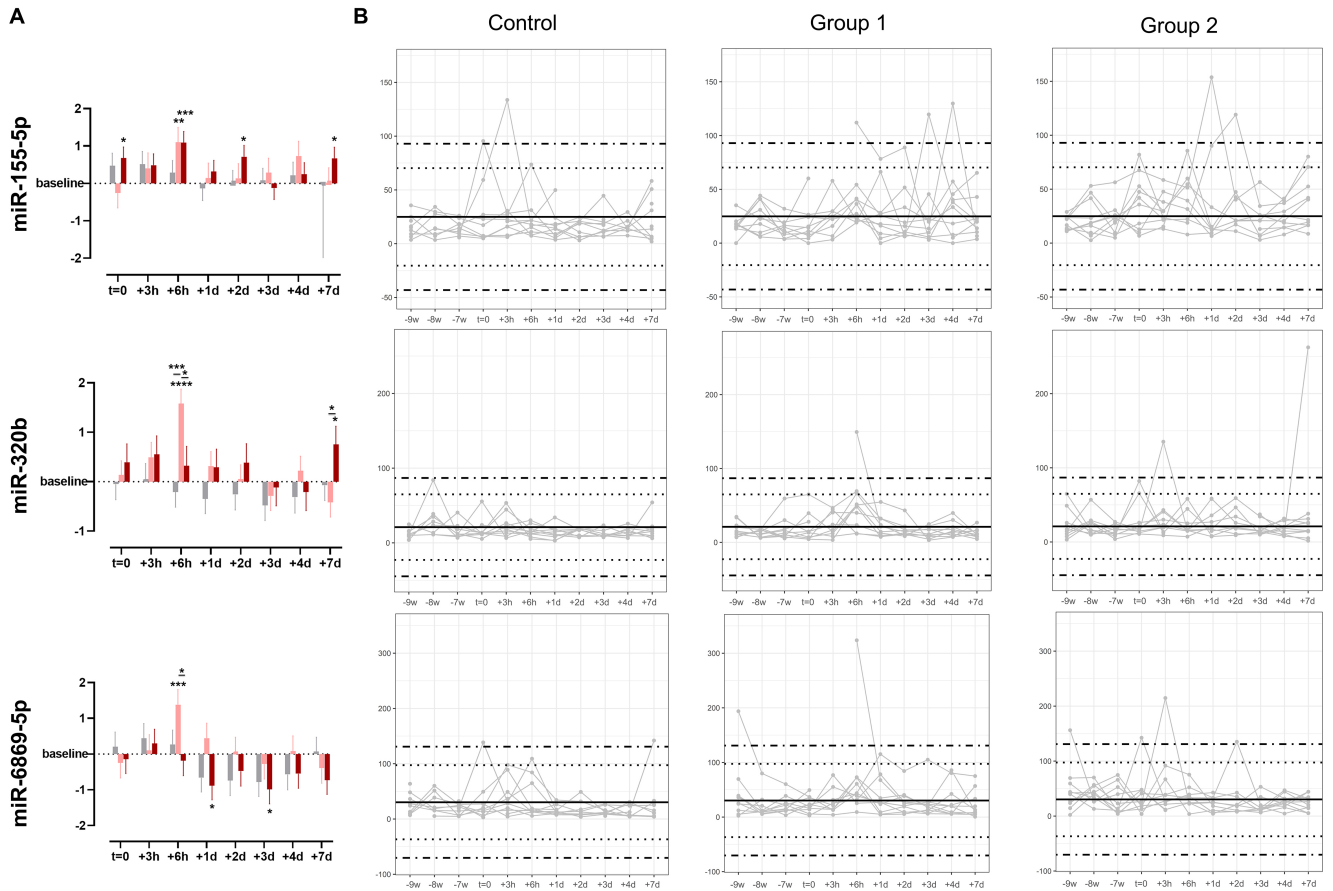
(A) Time-point specific differences in expression levels of selected miRNAs (miR-155-5p, miR-320b, and miR-6869-5p) given as log2FC. Data are shown as mean ± SE. Asterisks indicate significant differences in expression levels compared to baseline or treatment and control groups with adjusted *P*-values (two-way ANOVA: ^*^P < 0.05; ^**^P < 0.01; ^***^P < 0.001; and ^****^P < 0.0001), *n* = 10 per group. Gray color indicates control group, light red indicates Group 1, and dark red indicates Group 2; (B) Individual longitudinal profiles of regulated miRNAs. Data are shown as DESeq2-normalized reads per subject with *n* = 10 per group. Dotted lines depict z-scores of |2| and dot-dashed lines z-scores of |3|. ANOVA: Analysis of variance; miRNA: microRNA; log2FC: log2 fold change; SE: standard error; DESeq2: a normalization and differential expression analysis tool.

The time-point specific fluctuations and statistical differences of selected ABT-dependent miRNAs are depicted in Figure 6. miR-155-5p was significantly upregulated compared to baseline at *t* = 0, +6 h, +2 d, and +7 d in treatment group 2. In group 1, miR-155-5p was significantly upregulated only at +6 h. When comparing the log2FC of ABT-dependent regulation, no significant difference between the groups was detected at any of the time points. Considering the individual expression levels, most detected expression levels fell within the 99.7% probability threshold. However, two subjects in the control group appeared with expression levels exceeding the defined physiological threshold, which were interpreted as false positive results. Nevertheless, three and two subjects in groups 1 and 2, respectively, were detected as true positive results. Regarding miR-320b, an ABT-dependent initial upregulation until +2 d was observed in both treatment groups, whereas an overall downregulation was noted in the control group. Significant differences to baseline were detected in group 1 especially at +6 h, but also to control group and group 2. In group 2, time point +7 d was significantly different from baseline and from group 1. While the expression levels of none of the subjects in the control group exceeded the physiological range of the static model, one and two subjects were identified as truly ABT-doped in groups 1 and 2, respectively, due to exceeding the 99.7% probability threshold. When focusing on miR-6869-5p, an initial upregulation followed by a downregulation compared to baseline after one day (+1 d) was observed. This divergent direction of regulation was observed in all groups with a significant upregulation in group 1 at +6 h, and a significant downregulation compared to baseline in group 2 at +1 d and +3 d. While expression levels of two subjects in the control group only just exceeded the 99.7% threshold, one subject each in groups 1 and 2 showed expression levels exceeding the physiological threshold at baseline (-9 w). One and three further samples appeared outside the physiological range in groups 1 and 2, respectively.

## DISCUSSION

In the ever-lasting race between evolving doping modalities and ways to uncover them, indirect methods are the means of choice to detect blood doping. Thus, this study was designed as a proof-of-concept study to investigate the potential utility of the uEV-associated miRNA load to improve ABT detection.

Our observation of a highly significant increase in uEV excretion from 6 h until three days after ABT affirms the hypothesis that the re-transfusion of stored ECs boosts EV turnover and urinary excretion. In fact, EC storage promotes vesiculation^[22,23]^; however, most EVs originating from erythrocytes are cleared from the circulation within minutes; taken up by organs such as liver or kidney since EVs endeavor homeostasis in circulation^[69,70]^. Voss *et al.* reported that the highest concentration of erythrocyte-derived EVs in the circulation is reached 1 to 8 h after ABT^[71]^. Thus, the elevated post-ABT uEV concentration up to three days detected in this study seems to be an extended reaction of the body to the re-transfusion by re-distribution and creation of an overall balance between EV supply and excretion via urine. The higher SD at time points +2 d and +3 d compared to other time points may reflect a considerable biological variability to such an extended reaction. Apart from the EV-specific surface markers (i.e., CD63, CD9, and CD81), various other markers such as CD3, CD24, CD45, and CD133/1 were also observed abundantly in the study population in terms of occurrence or intensity. The biodistribution of re-transfused EVs or uEVs was beyond the scope of this study. However, considering that EVs are assumed to have a similar composition to their originating cells^[70]^, it can be anticipated that a majority of the uEV subset analyzed may originate from renal progenitor (CD133/1)^[72]^ and tubular epithelial cells (CD24)^[73]^, but also from T cells (CD3)^[74]^ and leukocytes (CD45)^[75]^. Driven by scientific interest and in addition to the overall characterization in the study population, we further evaluated uEV surface marker changes relative to baseline and between only the most distinct groups-control and group 2-to determine whether the increase in a specific uEV subpopulation could account for the significant rise in concentration following re-transfusion. However, this is not supported by the data as no significant inter- or intra-group differences were identified. MBFCM is a relative quantification method with a fixed set of capture and detection antibodies^[68]^. Hence, a proportion of the uEVs leading to the significantly increased concentration after ABT is likely to have evaded detection by MBFCM. In fact, three of the most abundantly detected surface markers (CD63, CD9, and CD133/1) show a trend of downregulation in the ABT group while the control group seems to be upregulated, possibly as a consequence of the longitudinal sampling schedule. This trend may contribute to the overall discriminative pattern between the groups observed in cluster analysis.

While the uEV characterization provided valuable insights, the focus of the present study was not on using uEV surface markers as indicators of ABT, but rather on their associated miRNA cargo. Processing of separated uEVs yielded high-quality small RNAs for successful sequencing, which is in line with results reported elsewhere^[51]^. Further, downstream transcriptomic analyses revealed that ABT alters uEV-associated miRNA cargo as emphasized by 13 miRNAs significantly differently expressed in both groups receiving ABT (group 1 and group 2) but not the control group. The largest and most significant effect was shown for the most abundant three differently expressed miRNAs 6 h after ABT (miR-155-5p, miR-320b, and miR-6869-5p). In real-world anti-doping testing, the time frame of doping is completely unknown and testing would be performed irrespective of an athlete’s state of recovery from a previous blood donation. This is why an individual’s profile of hematological markers is monitored longitudinally via the ABP^[7]^. Any value that exceeds the individual’s physiological range would indicate potential doping. As proof-of-concept, a group-wise longitudinal profile was generated for each of the three uEV-derived miRNAs, revealing an overall high specificity but low sensitivity for all three (miR-155-5p: 2.84% sensitivity, 98.61% specificity; miR-320b: 1.70% sensitivity, 100% specificity; miR-6869-5p: 2.27% sensitivity, 97.22% specificity). Thus, the potential of these single uEV-derived miRNAs to detect suspicious patterns over time appears modest due to the subtle nature of the ABT-induced changes. As part of our comprehensive analysis, we performed sparse partial least squares discrimination analysis (sPLS-DA) to assess the most predictive uEV-miRNA patterns for ABT discrimination as described in detail elsewhere to ensure all relevant factors were considered^[76]^. The results were in line with expectations and did not contribute meaningfully to the broader research perspective. In the interest of focusing on the more relevant and significant findings and preventing potential misinterpretation or over-interpretation of results, the data from these analyses were deemed negligible to report. As already discussed by other researchers and compiled by Mussack *et al.*^[43,76]^, inter- and intra-individual molecular variations introduce a huge challenge in ABT detection, as do the use of masking agents and the use of micro-dosing fresh *vs*. frozen or cryo-preserved blood products, and many other factors. Despite these limitations, three uEV-derived miRNAs (miR-155-5p, miR-320b, and miR-6869-5p) were observed to be significantly regulated after ABT, supporting their physiological relevance. ABT leads to a short-term increase in blood volume and, thus, a rise in blood pressure triggering its regulation via the renin-angiotensin-aldosterone system (RAAS). miR-155 targets the angiotensin-II-type-1-rezeptors^[77]^. Hence, an increased miR-155 level, as observed in uEVs after ABT, may contribute to RAAS inhibition, leading to a normalization of the volume load including decreasing blood pressure and increasing diuresis. In contrast to miR-155, miR-6869-5p has an activating effect on RAAS^[78]^. Thus, its observed downregulation in uEVs after ABT could have a supportive effect on blood-pressure regulation. The presence of miR-155 and miR-6869-5p in uEVs suggests that once volume homeostasis has been achieved, any excessive miRNAs may be excreted via the kidney and urine. Despite its involvement in RAAS regulation, miR-155 is also known to function as a marker for urologic malignancies^[79]^. Its involvement in immune and inflammatory responses^[80,81]^, along with its upregulation during erythropoiesis^[82]^, was also reported. Urinary miR-320b and miR-6869-5p are also known for their relevance in kidney disorders^[78,83]^. Even though pathways of erythrocyte differentiation and homeostasis are significantly enriched with targets of miR-320b^[84]^, there is currently no experimental study that establishes a direct link of miR-320b with erythropoietic or diuretic processes.

Nevertheless, this proof-of-concept study was rigorously designed, analytically ambitious using a broad range of qualitative and quantitative methods and executed in compliance with MISEV guidelines^[41,42]^. However, our study with the existing group sizes, experimental setup and urine sampling regimen also had its limitations, e.g., the inter- and intra-individual urine quantity, quality and creatinine levels, the comparison of stored *vs*. frozen or cryo-preserved urine or uEVs isolates, the individual secretion dynamics and kinetics of uEVs after ABT, including their molecular variations, and many other factors as discussed earlier^[43,76]^. However, the implementation of uEV quantity and molecular markers in routine anti-doping testing seems premature and further research is required to verify and develop reliable test systems and advanced biomarker signatures. For example, the specific uEV subpopulation that contributes to the increase in concentration after ABT could be identified, and their associated miRNA profiles could be subsequently analyzed.

## CONCLUSION

Overall, we demonstrated in this present feasibility study that uEV separation via immunoaffinity yields sufficient material for successful downstream vesicle characterization. We revealed not only typical vesicular structures and proteins, but also gained an overview of potential ABT-dependent surface marker patterns which, in addition, appear to indicate a leukocytic origin of the studied uEVs. uEV enumeration allowed us to confirm that the re-transfusion of stored ECs indeed results in significantly increased uEV excretion. In addition, significantly altered expression levels of a uEV-associated set of miRNAs are observed, indicating their regulatory impact on RAAS.

In summary, we demonstrated that the molecular composition in uEVs displays modest changes after ABT on the surface marker and miRNA level, thus contributing to the general understanding of uEV composition and the effect of ABT on uEV-associated surface markers and miRNAs.

## DECLARATIONS

### Availability of data and materials

We have submitted all relevant data of our experiments to the EV-TRACK knowledgebase (EV-TRACK ID: EV250063)^[67]^. Raw sequence reads acquired by small RNA-Seq experiments are deposited at the European Nucleotide Archive (study accession number PRJEB89432).

### Copyright

© The Author(s) 2025.
