# Supplementary material for: Impact of autologous blood transfusions on surface marker and microRNA profiles of urinary extracellular vesicles
Source: Extracell Vesicles Circ Nucl Acids. 2025 Nov 28;6(4):876–94. doi: 10.20517/evcna.2025.89 (PMC12809685; doi:10.20517/evcna.2025.89)
Supplement: Supplementary file 1 [file evcna-6-4-876-SupplementaryMaterials.pdf]

Supplementary Materials

Impact of autologous blood transfusions on surface marker and microRNA profiles of urinary extracellular vesicles

Veronika Mussack, **Michael W. Pfaffl**

Animal Physiology and Immunology, School of Life Sciences Weihenstephan, Technical University of Munich, Freising 85354, Germany.

Correspondence to: Prof. Michael W. Pfaffl, Animal Physiology and Immunology, School of Life Sciences Weihenstephan, Technical University of Munich, Freising 85354, Germany. E-mail: michael.pfaffl@tum.de

Supplemental Figure 1. Western blot analyses. (A) Ponceau S staining of total protein composition. (B) Murine immunoglobulin G background signals.

Supplemental Figure 2. Western blot analyses, uncropped images.

Supplemental Figure 3. Multiplexed bead-based phenotyping. (A) Cell-free urine only. (B) Negative control.

Supplemental Figure 4. Representative electropherogram of uEV-associated RNA.

Supplemental Table 1. Antibodies utilized in western blot analyses.

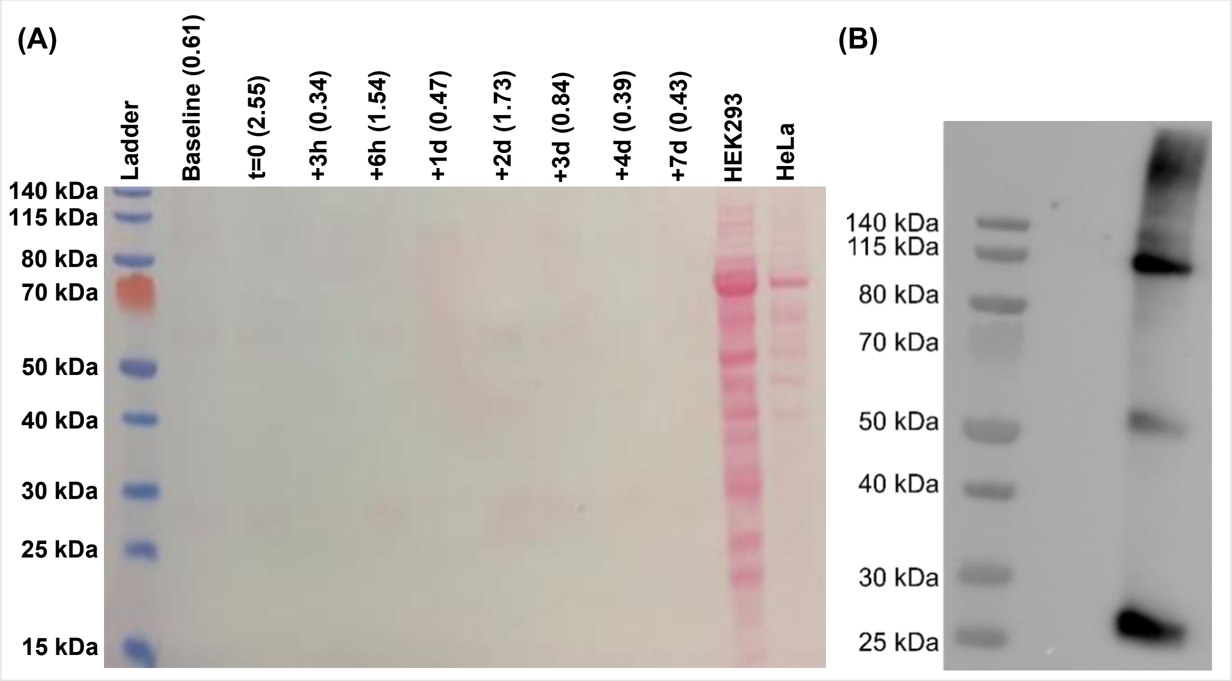

Supplemental Figure 1. Western blot analyses. (A) Ponceau S staining of total protein composition. The number in brackets indicates each sample's initial urinary creatinine concentration in [g/l]. (B) Murine immunoglobulin G (mIgG) staining as background control. CD9+/CD63+/CD81+ EVs were positively selected by magnetic beads containing mIgG. Western blot membrane was incubated with secondary anti-mouse antibody only to detect western blot bands specific to the capture antibodies and not to the samples. Supplemental Figure 1B is reprinted from: Comparing small urinary extracellular vesicle purification methods with a view to RNA sequencing—Enabling robust and non-invasive biomarker research; Biomolecular Detection and Quantification; Vol 17; Authors: Veronika Mussack, Georg Wittmann, Michael W. Pfaffl; Pages No. 100089; https://doi.org/10.1016/j.bdq.2019.100089; Copyright (2019), with permission from Elsevier and the authors; RightsLink license number 5011841392049.

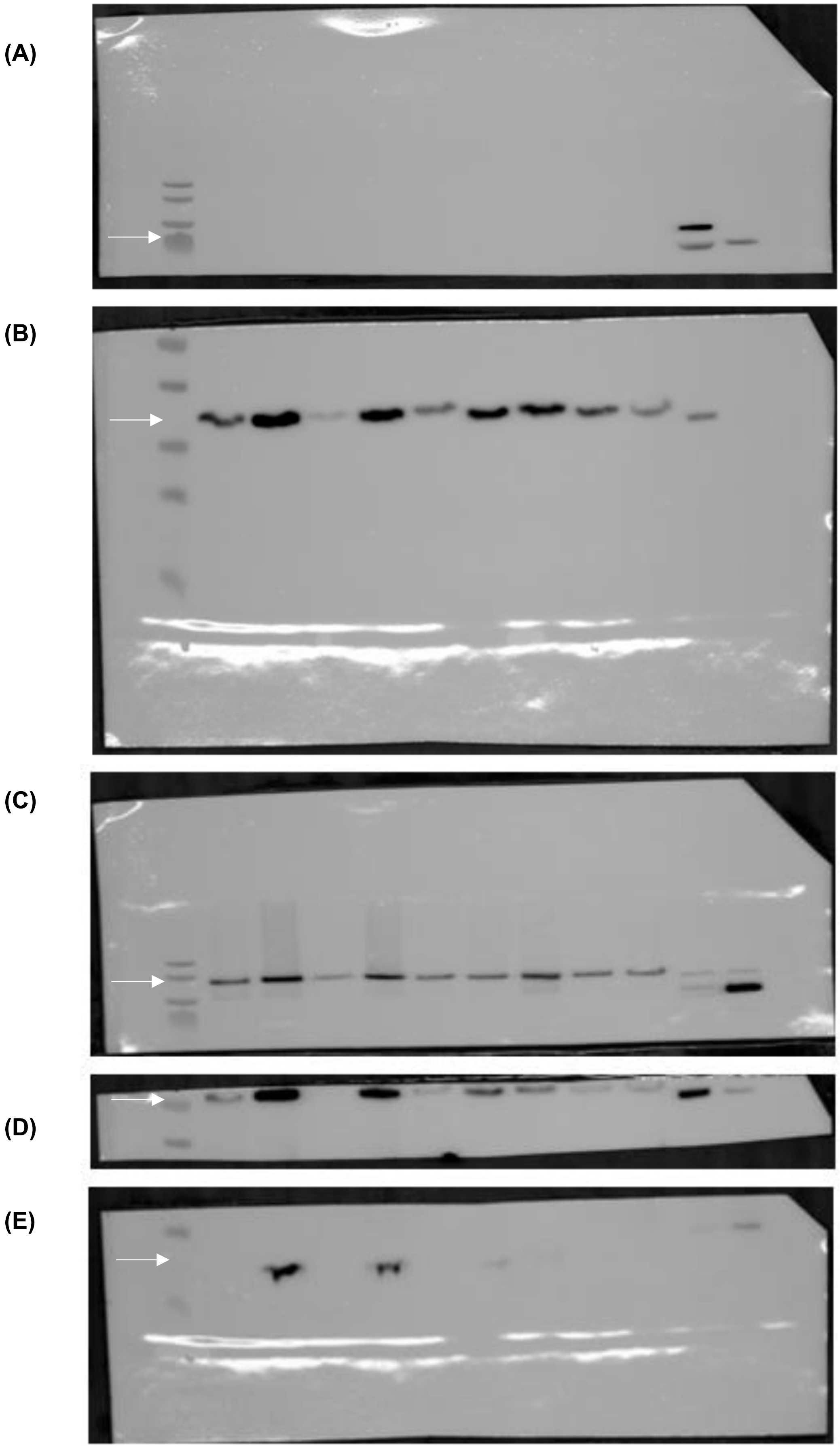
(A)
(B)
(C)
(D)
(E)

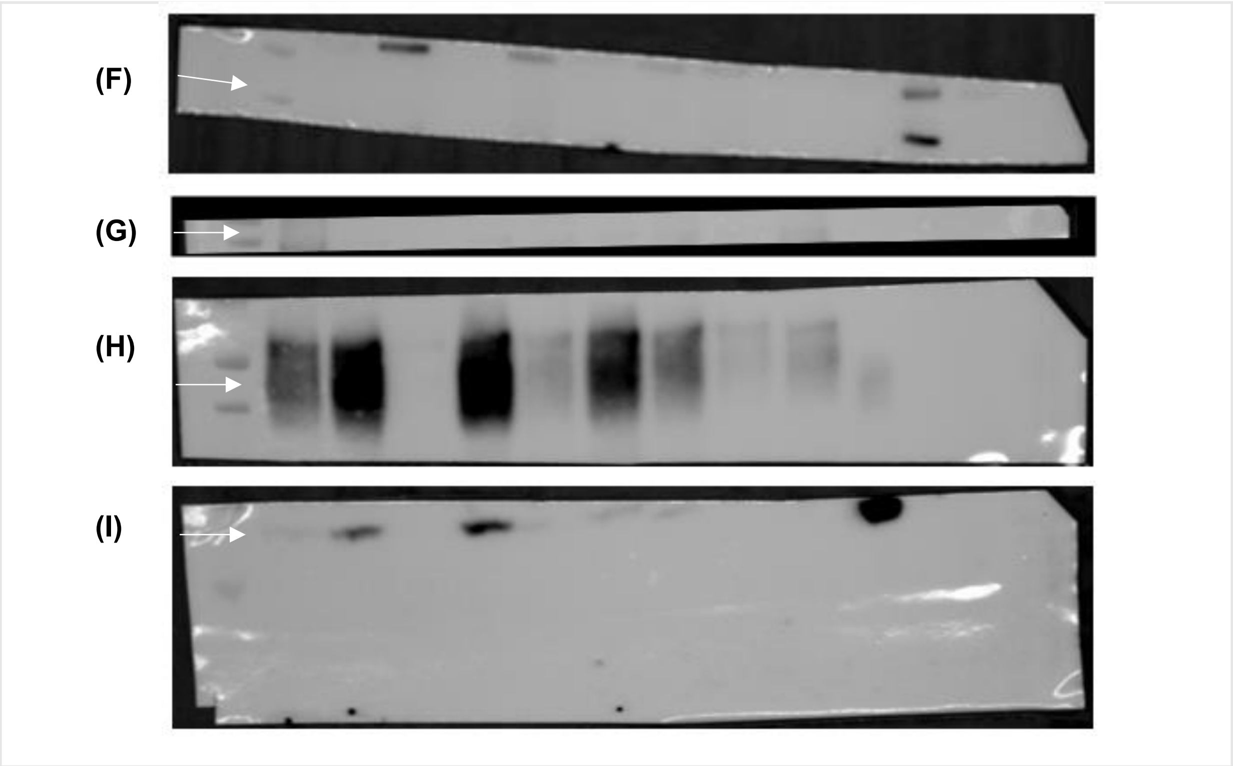

Supplemental Figure 2. Western blot analyses, uncropped images. (A)-(B) Membrane one in reducing conditions, first day. (A) Top section (size range: ~60kDa to >140kDa) incubated with anti-Calnexin antibody. (B) Bottom section (size range: <15kDa to ~60kDa) incubated with anti-Syntenin antibody. (C)-(E) Membrane one in reducing conditions, second day. (C) Top section (size range: ~60kDa to >140kDa) incubated with anti-Alix antibody. (D) Mid-section (size range: ~35kDa to ~60kDa) incubated with anti-TSG101 antibody. (E) Bottom section (size range: <15kDa to ~35kDa) incubated with anti-CD9 antibody. (F) Membrane one in reducing conditions, third day. Mid-section (size range: ~35kDa to ~60kDa) incubated with anti-EPCAM antibody. (G)-(I) Membrane two in non-reducing conditions, first day. (G) Top section (size range: ~75kDa to ~115kDa) incubated with anti-Uromodulin antibody. (H) Mid-section (size range: ~35kDa to 70kDa) incubated with anti-CD63 antibody. (I) Bottom-section (size range: <15kDa to 25kDa) incubated with anti-CD81 antibody. (*) uEVs were positively selected using magnetic beads coated with mIgG. Owing to the high signal intensities of the mIgG background, cutting off the respective size ranges was necessary to maximize exposure time and capture signals for markers with lower signal intensities. Extensive pre-experiments enabled us to determine appropriate horizontal cut lines without losing or obscuring information from western blot analyses. Cutting was performed prior to antibody incubation.

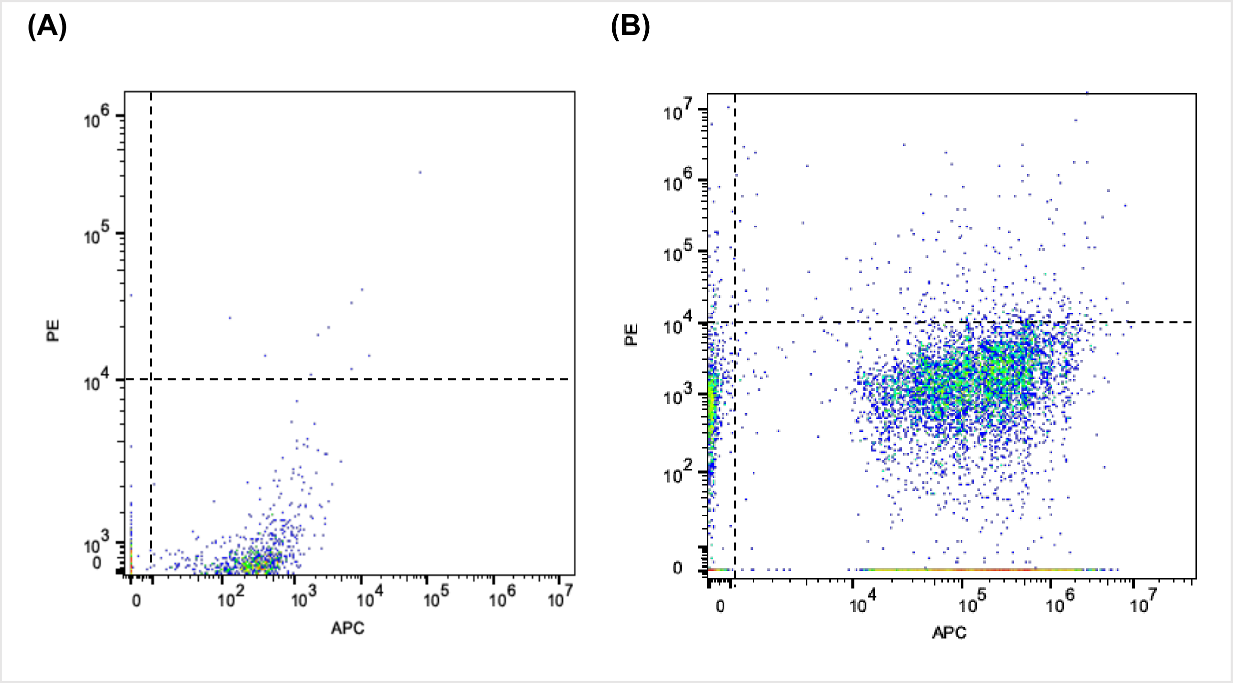

Supplemental Figure 3. Multiplexed bead-based phenotyping. (A) Cell-free urine only. FITC vs. PE exhibited no detectable signal. (B) Negative control. Blank with only capture beads and MACSPlex buffer.

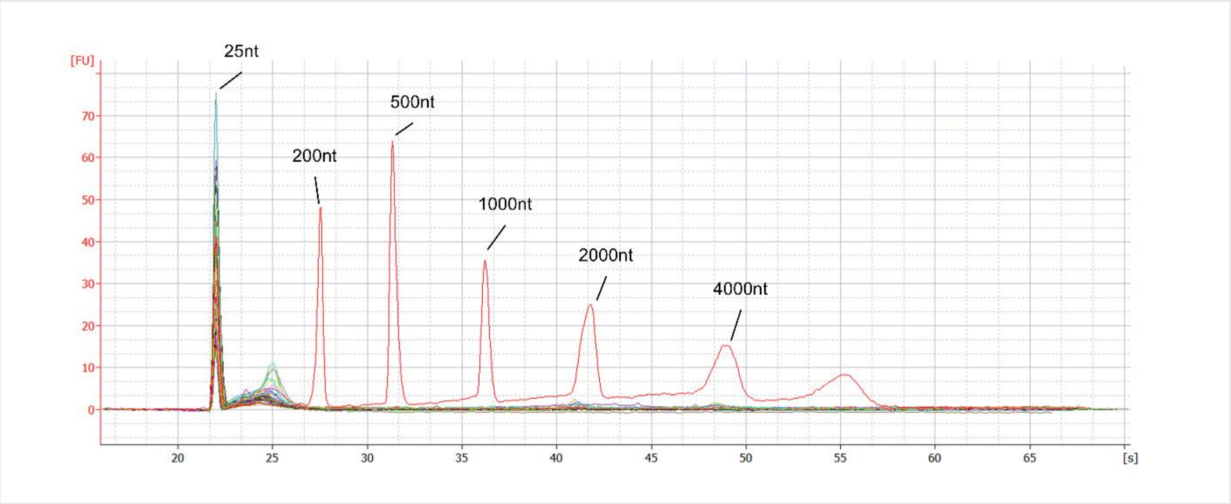

Supplemental Figure 4. Representative electropherogram of uEV-associated RNA. The electropherogram shows and overlay of all samples analyzed (n=321) using the RNA 6000 Pico Kit and the Bioanalyzer 2100 (Agilent Technologies, Germany). The prominent red lines indicate the ladder.

Supplemental Table 1. Antibodies utilized in western blot analyses

**Primary antibodies**		
goat anti-Calnexin	WA-AF1179a, Biomol	1:2,500 in blocking buffer
mouse anti-Uromodulin	Clone B-2, sc-271022, Santa Cruz	1:100 in blocking buffer
rabbit anti-Alix	Clone EPR15314-33, ab186728, Abcam	1:1,000 in blocking buffer
rabbit anti-HSP70	Clone EPR17677, ab182844, Abcam	1:1,000 in blocking buffer
rabbit anti-Syntenin	Clone EPR8102, ab133267, Abcam	1:5,000 in blocking buffer
mouse anti-TSG101	Clone 4A10, ab83, Abcam	1:800 in blocking buffer
rabbit anti-EPCAM	ab32392, Abcam	1:1,000 in blocking buffer
mouse anti-CD63	Clone TS63, ab59479, Abcam	1:500 in blocking buffer
mouse anti-CD81	Clone M38, ab79559, Abcam	1:833 in blocking buffer
rabbit anti-CD9	Clone EPR2949, ab92726, Abcam	1:1,000 in blocking buffer
**Secondary antibodies, conjugated to horseradish peroxidase**		
goat anti-Mouse	ab97040, Abcam	1:6,666 in blocking buffer
goat anti-Rabbit	ab97080, Abcam	1:6,666 in blocking buffer
rabbit anti-Goat	ab97105, Abcam	1:6,666 in blocking buffer
